# Supplementary material for: Bayesian refinement of protein structures and ensembles against SAXS data using molecular dynamics
Source: PLoS Comput Biol. 2017 Oct 18;13(10):e1005800. doi: 10.1371/journal.pcbi.1005800 (PMC5662244; doi:10.1371/journal.pcbi.1005800)
Supplement: S1 Archive — (BZ2) [file pcbi.1005800.s011.tar.bz2 › gromacs_4.62_bayesian_saxs_refi/share/html/online/g_sgangle.html]

g\_sgangle


| g\_sgangle Main Table of Contents |

**VERSION 4.6  
Sat 19 Jan 2013**

---

### Description

Compute the angle and distance between two groups.
The groups are defined by a number of atoms given in an index file and
may be two or three atoms in size.
If -one is set, only one group should be specified in the index
file and the angle between this group at time 0 and t will be computed.
The angles calculated depend on the order in which the atoms are
given. Giving, for instance, 5 6 will rotate the vector 5-6 with
180 degrees compared to giving 6 5.

If three atoms are given,
the normal on the plane spanned by those three atoms will be
calculated, using the formula P1P2 x P1P3.
The cos of the angle is calculated, using the inproduct of the two
normalized vectors.

Here is what some of the file options do:  
-oa: Angle between the two groups specified in the index file. If a group contains three atoms the normal to the plane defined by those three atoms will be used. If a group contains two atoms, the vector defined by those two atoms will be used.  
-od: Distance between two groups. Distance is taken from the center of one group to the center of the other group.  
-od1: If one plane and one vector is given, the distances for each of the atoms from the center of the plane is given separately.  
-od2: For two planes this option has no meaning.

### Files

| option | filename | type | description |
| --- | --- | --- | --- |
| **-f** | traj.xtc | Input | Trajectory: xtc trr trj gro g96 pdb cpt |
| **-n** | index.ndx | Input | Index file |
| **-s** | topol.tpr | Input | Run input file: tpr tpb tpa |
| **-oa** | sg\_angle.xvg | Output | xvgr/xmgr file |
| **-od** | sg\_dist.xvg | Output, Opt. | xvgr/xmgr file |
| **-od1** | sg\_dist1.xvg | Output, Opt. | xvgr/xmgr file |
| **-od2** | sg\_dist2.xvg | Output, Opt. | xvgr/xmgr file |

### Other options

| option | type | default | description |
| --- | --- | --- | --- |
| **-[no]h** | bool | no | Print help info and quit || **-[no]version** | bool | no | Print version info and quit || **-nice** | int | 19 | Set the nicelevel || **-b** | time | 0 | First frame (ps) to read from trajectory || **-e** | time | 0 | Last frame (ps) to read from trajectory || **-dt** | time | 0 | Only use frame when t MOD dt = first time (ps) || **-[no]w** | bool | no | View output .xvg, .xpm, .eps and .pdb files || **-xvg** | enum | xmgrace | xvg plot formatting: xmgrace, xmgr or none || **-[no]one** | bool | no | Only one group compute angle between vector at time zero and time t || **-[no]z** | bool | no | Use the z-axis as reference |

---

http://www.gromacs.org  
gromacs@gromacs.org
